# Calcium Phosphate-Coated Lipid Nanoparticles as a Potential Tool in Bone Diseases Therapy

**DOI:** 10.3390/nano11112983

**Published:** 2021-11-06

**Authors:** Simona Sapino, Giulia Chindamo, Daniela Chirio, Maela Manzoli, Elena Peira, Chiara Riganti, Marina Gallarate

**Affiliations:** 1 Department of Drug Science and Technology, University of Torino, Via P. Giuria 9, 10125 Torino, Italy; simona.sapino@unito.it (S.S.); giulia.chindamo@unito.it (G.C.); maela.manzoli@unito.it (M.M.); elena.peira@unito.it (E.P.); marina.gallarate@unito.it (M.G.); 2 Department of Oncology, University of Torino, Via Santena 5/bis, 10126 Torino, Italy

**Keywords:** lipid nanoparticles, calcium phosphate coating, bone diseases, human osteosarcoma cells, cellular uptake

## Abstract

The treatment of bone diseases (including osteoporosis, osteoarthritis, and bone cancer) often results in reduced efficiency and/or adverse reactions due to the fact that it is not specifically targeted to the site of action. The employment of a suitable carrier should increase drug location to the site of bone disease. The purpose of this study is to prepare and characterize lipid nanoparticles (NPs) coated with calcium phosphate (CaP-NPs). A coating method, to date used only to obtain liposomes covered with CaP, is herein partially-modified to prepare CaP-coated lipid NPs. An extensive physico-chemical characterization was achieved by employing several techniques (DLS, SEM and TEM, and both combined with EDS, XRD, and FTIR) that confirmed the feasibility of the developed coating method. Preliminary uptake studies on human osteosarcoma cells (U-2OS) were performed by entrapping, as a lipid probe, Sudan Red III in NPs. The obtained data provided evidence that CaP-NPs showed higher cell accumulation than uncoated NPs. This result may have important implications for the development of drug loaded CaP-NPs to be tested in vitro with a view of planning future treatment of bone diseases, and indicate that CaP-NPs are potential vehicles for selective drug delivery to bone tissue.

## 1. Introduction

Due to their similarity to bone matrix, CaP materials (particulates, ceramics, cements, and scaffolds) have recently received a lot of research attention in both orthopedic and dental implants due to their biocompatibility and their ability to bond directly to bone [1]. In addition, basing on data reported in the literature, these inorganic materials have potential for local delivery to bone sites [2].

Depending on their composition, CaP systems are classified as calcium hydroxyapatite (HA), alpha- or beta-tricalcium phosphate (α- or β-TCP), biphasic calcium phosphates (BCPs) for mixtures of HA and β-TCP, and unsintered apatites [3]. They can promote osteoblast adhesion and proliferation, osseointegration, and deposition of calcium-containing minerals. Several researchers described the application of nanoscale CaP coatings on implants to enhance their bioactivity and to facilitate osseointegration with natural bone. The different compositions (e.g., HA, brushite or apatite), crystallinity (amorphous or crystalline state) and surface features of the substrate (e.g., polished or roughened) can all affect osseointegration in vivo [4], and extensive research has been described in the literature on different CaP deposition techniques [5]. Besides these, nano-CaP crystals with different structures including particles, spheres, rods, needles, wires, fibers, and disks/flakes/platelets/strips have been fabricated using various synthetic methods, to achieve better bone regeneration than by using conventional CaP biomaterials [4].

As regards drug delivery, nanotechnologies (from NPs to nanostructured scaffolds) have been widely studied to increase efficacy and specificity with respect to the treatment of bone diseases, which include cancer bone metastasis, osteosarcoma, bone infections and inflammatory diseases, osteoarthritis, and bone regeneration. They offer the advantages of protecting the cargo, increasing its retention time and carrying the drug to its destination achieving a specific delivery via surface modification [6].

Several types of NPs, either organic or inorganic, are currently under study [7] for the treatment of bone diseases. Organic NPs typically include polymer (e.g., poly[lactic-co-glycolic acid] PLGA) based NPs. On the other hand, inorganic NPs mainly include silica-based mesoporous NPs and layered double hydroxides (LDH). Drugs delivered for bone diseases include those traditionally used such as antibiotics and chemotherapeutics, and gene therapy reagents such as plasmid deoxyribonucleic acid (DNA) or small interfering ribonucleic acid (siRNA) [6]. More recently, bisphosphonates (BPs) have been proposed for treating bone diseases. In vivo and in vitro studies demonstrate that BPs inhibit bone resorption through the reduction of osteoclast activity and differentiation and enhance osteoblast differentiation [8]. Since they also act as affinity agents, they are grafted onto NPs for the specific delivery of other drugs to bone tissues [9].

Wang et al. [10], taking advantage of the strong mineral affinity of BPs, developed BP-functionalized liposomes and micelles that showed sustained release in in vitro studies and enhanced retention in mineralized collagen scaffolds in an animal implant model. These BP-decorated nanocarriers allowed better control of local drug delivery in specific diseases where local administration of mineral-binding nanocarriers is beneficial. A study of Salamanna et al. suggested that the use of alendronate-functionalized HA nanocrystals could represent a promising strategy especially in osteoporosis patients with high risks of spinal fusion failure [11].

In the last few years, few attempts have been made to prepare CaP-coated nanostructures that could be very useful in bone bio-applications as ion exchangers, catalysts, scaffolds, and as drug delivery systems. Schmidt et al. [12] described a nanoshell synthesis strategy to form aqueous-cored CaP-NPs that consists in depositing nanometers-thick coatings on dioleoyl phosphatidic acid (DOPA) liposomes. Using a simplified batch reaction strategy, they improved the long-term stability of the particle suspensions and were able to modulate both particle size and coating thickness.

Similarly, Yeo et al. [13] developed CaP nanoshells by employing different liposomes (DOPA and diphenyl phosphoryl azide (DPPA)) as templates. CaP nanoshells were demonstrated to be partially crystalline or amorphous in structure. The particle size distribution analysis showed that the CaP nanoshells produced using the DOPA template were more monodispersed than those produced using the DPPA template.

More recently, Rivero Berti et al. [14] examined the delivery capacity and mechanical stability of CaP-coated-DOPA liposomes and demonstrated their close interaction with *Staphylococcus aureus*, suggesting their potential employment as a drug delivery system to control biofilm growth.

Despite this interest, except for liposomes, to the best of our knowledge no one has so far proposed CaP-coated lipid NPs to be used as drug delivery systems for bone diseases.

Owing to their superior biocompatibility and kinetic and physical stability, lipid NPs have been widely applied as nanocarriers for drug delivery [15]. Given their high versatility, lipid NPs have been widely proposed for oral delivery [16] but they have been extensively used also for topical [17] purposes, studied for parenteral applications [18], and more recently, used for pulmonary and ocular administration [15] too.

The main aim of the present study is to prepare novel CaP-coated lipid NPs (CaP-NPs) by developing and optimizing a simple and reliable method that exploits the charged surface of the lipid matrix to allow the localization of calcium and phosphate ions onto NPs’ surface. Furthermore, to assess the potential ability of CaP-NPs to deliver drugs in bone tissues, CaP-NPs are loaded with Sudan Red III, chosen as a lipid probe. Human osteosarcoma cells are exposed to the dye-loaded NPs to evaluate the influence of CaP-coating on the cellular uptake in order to plan future studies on drug-loaded CaP-NPs (which will be the topic of a soon forthcoming paper).

## 2. Materials and Methods

### 2.1. Chemicals

Trilaurin, benzyl alcohol, ethyl acetate, propylene glycol, Sudan Red III (1-[4-(phenylazo)phenylazo]-2-naphthol), sodium hydroxide, calcium chloride, sodium phosphate dibasic dihydrate, myristic acid, stearic acid, behenic acid, taurocholic acid sodium salt (TC), stearylamine, Sepharose^®^CL4B were obtained from Sigma Aldrich (St. Louis, MO, USA). Epikuron^®^200 (soybean lecithin, containing 95% phosphatidylcholine) was obtaine from Cargill (Minneapolis, MN, USA); Cremophor^®^RH60 (PEG-60 hydrogenated castor oil) was from Acef (Piacenza, Italy); sodium chloride and phosphoric acid were from Alfa Aesar (Haverhill, MA, USA).

### 2.2. Preparation of NPs and Screening of Charging Agents

Lipid NPs (hereafter defined as NPs) were prepared following our previously developed method called “cold microemulsion dilution” [19,20]. Firstly, an oil-in-water microemulsion (µE) was obtained by mixing 200 µL water-saturated EA (s-EA) with appropriate amounts of surfactant, cosolvent, and cosurfactant and then adding, drop by drop, 700 µL EA-saturated water (s-water) to the lipid phase by vortexing at room temperature up to transparency. A solution of trilaurin in s-EA was used as lipid phase, while Epikuron^®^200, Cremophor^®^RH60, benzyl alcohol and propylene glycol were employed as surfactant/cosurfactant. Afterwards, the thus prepared µE was diluted with 5 mL water at room temperature to induce the diffusion of the organic solvent from the internal phase into the added water, which determines the precipitation of NPs.

As the presence of a charge on NPs surface is necessary to ensure the coating process, a selection of different charging agents has been carried out in order to obtain suitable surface properties. Particularly, either a negative (TC or fatty acid) or a positive (stearylamine) charging agent has been used to obtain different µEs, that, upon dilution with 5 mL water, gave rise to differently charged NPs (NP_NEG_ or NP_POS_, respectively). The compositions of the developed µEs are detailed in Table 1.

### 2.3. Purification of NPs

0.5 mL of freshly prepared NPs underwent gel filtration using a matrix of cross-linked agarose (Sepharose^®^CL4B) as the stationary phase and 0.3 M NaCl as the mobile phase. The resulting opalescent fraction (3 mL) containing the purified NPs was then dialyzed overnight at room temperature against ultrapure water with a MWCO of 14,000 Da to remove the excess salts and unentrapped molecules.

### 2.4. Characterization of NPs

#### 2.4.1. Particle Size and Zeta Potential Measurements

Mean diameters of both NPs and CaP-NPs were measured in triplicate using dynamic light scattering (DLS, Brookhaven Instruments, Holtsville, NY, USA). Zeta potential was measured after proper dilution of NPs dispersions, through the same instrument using the Zeta potential mode by the average of 10 measurements.

#### 2.4.2. SEM and FESEM Characterization

Morphology and size were determined via Scanning Electron Microscopy (SEM-Stereoscan 410, Leica Microsystems, Wetzlar, Germany) and Field Emission (FE)SEM (Tescan S9000G FESEM 3010 microscope working at 30 kV, equipped with a high brightness Schottky emitter). For analyses, some drops of either NPs or CaP-NPs were placed on an aluminum stub coated with a conducting adhesive and left to dry under vacuum for one night. Both instruments are fitted with Energy Dispersive X-ray Spectroscopy (EDS) analysis.

#### 2.4.3. TEM Characterization

Transmission electron microscopy (TEM) observations were performed via JEOL JEM 3010-UHR (Peabody, MA, USA) microscope operating at 300 kV and equipped with an Oxford Inca Energy TEM 200 EDS X-rays analyzer. For the measurements, samples were prepared by dripping on a carbon-coated copper grid and water was evaporated in air.

### 2.5. CaP Coating

Basically, two different methods of CaP coating have been developed: method A and method B. Method A was applied directly to unpurified NP_POS_, whereas method B was applied both to NP_POS_ and to NP_NEG_ previously purified via gel filtration.

- **Method** **A**

CaP coating of NP_POS_ was performed properly adapting a method reported in the literature for coating liposomes [12]: 1 mL 69 mM CaCl_2_ and 1 mL 41 mM H_3_PO_4_ aqueous solutions were added in a single aliquot to 0.5 mL or 1 mL of NP_POS_2 suspension; the pH was adjusted to 8.5 with 1M NaOH and monitored using a pHmeter (Sension+ PH3, Hach Lange, Lainate, Italy) and the mixture was finally magnetically stirred for different times: 120, 180, or 240 min (Table 2). The obtained coated NPs were indicated as CaP-NP_POS_2 (A-1 to A-5).

- **Method** **B**

An aliquot (0.5 mL) of NPs was purified via gel filtration to obtain 3.0 mL of NP aqueous suspension that was successively dialyzed overnight at room temperature against ultrapure water with a 14,000 Da MWCO to eliminate Na^+^ and Cl^−^ ions introduced during gel filtration. Samples were further 1:2 diluted with ultrapure water. Afterwards, 300 μL 42 mM CaCl_2_ then 300 μL 42 mM Na_2_HPO_4_, then 300 μL 42 mM CaCl_2_ and finally 300 μL 42 mM Na_2_HPO_4_ were added with this layer-by-layer sequence to NP_NEG_1 suspensions adapting a method reported in the literature [21]. For NP_POS_2 the first addition of CaCl_2_ was avoided. After each addition, the mixture was magnetically stirred for 20 min. During the coating procedure, the pH was monitored and maintained at 8.5 in order to ensure two negative charges on the phosphate group. The obtained coated NPs were indicated as CaP-NP_POS_2B and CaP-NP_NEG_1B. The resulting suspensions of CaP-NPs were then dialyzed overnight at room temperature against ultrapure water with a 14,000 Da MWCO to separate the excess ions.

### 2.6. Coating Evaluation

#### 2.6.1. Particle Size and Zeta Potential Measurements

In order to verify the deposition of CaP on the NPs surface, particle size and Zeta potential were monitored after addition of each layer of the CaP coating. Analyses were performed following the procedure reported in Section 2.4.1.

#### 2.6.2. SEM-EDS and TEM-EDS Analyses

To check the presence of the CaP coating, both the uncoated and the CaP-coated NPs were analyzed via energy-dispersive spectroscopy coupled with SEM and FESEM (SEM–EDS), and with TEM (TEM–EDS) to detect calcium and phosphorus. Details on the instruments were described in Section 2.4.2 and Section 2.4.3.

#### 2.6.3. X-ray Diffraction

Powder X-ray diffraction (XRD) patterns were collected via X’Pert Pro Bragg Brentano diffractometer (Philips) using CuKα radiation (λ = 1.5406 Å) operating at 30 mA and 40 kV, in the range of 2θ = 5–60°, step size 0.015°, time per step 33 s.

#### 2.6.4. FTIR Spectroscopy

Fourier transform infrared spectra (FTIR) were recorded via JASCO FTIR-5300 working with resolution of 4 cm^−1^ over 64 scans. Some drops of each sample were deposited on a silicon platelet, left to dry and placed in a quartz cell equipped with KBr windows, designed for RT studies in vacuum and controlled atmosphere. Before FTIR analysis, the samples were outgassed at room temperature to remove physically adsorbed water and impurities.

### 2.7. Stability Studies

#### 2.7.1. Size and Zeta Potential

To assess the stability of the coated NPs, DLS analysis and Zeta potential determinations of CaP-NP_POS_2 and CaP-NP_NEG_1 were carried out at periodic intervals over a six-week period, storing the samples at 4 °C and following the procedure described in Section 2.4.1.

#### 2.7.2. Freeze-Drying/Re-Suspension of NPs

Long-term storage of CaP-NPs was assayed via freeze-drying either in the absence or in the presence of a cryoprotectant (10% *w*/*v* trehalose or glucose). For this purpose, CaP-NPs suspensions, without or with cryoprotectant, were stored at −40 °C overnight and then dried under 50 mtorr atmosphere for 24 h. Samples were then reconstituted in milliQ water under agitation. Mean diameter, polydispersity index, and Zeta potential were then determined on the reconstituted samples.

### 2.8. Incorporation of Sudan Red III and Determination of Entrapment Efficiency

Sudan Red III-loaded NPs were prepared by introducing 1 mg dye in the lipophilic phase of the selected NPs. The entrapment efficiency (%EE) was evaluated via HPLC that determines Sudan Red III concentration after and before gel filtration with Sepharose^®^CL4B. More precisely, the unpurified NPs suspension (pre-column) and the fraction that contains purified NPs, eluted through gel filtration (post column), were analyzed after proper dilution. EE% was determined as the ratio between the amount of dye in the post-column fraction and that in the pre-column suspension ×100. HPLC analysis was performed using a LC9 pump (Shimadzu, Tokyo, Japan) with an Teknokroma^®^ ODS- 5 µm 15 cm × 0.46 column and a C-R5A integrator (Shimadzu, Tokyo, Japan); detector: UV-Vis λ = 520 nm (Shimadzu, Tokyo, Japan). Sudan Red III was eluted in isocratic conditions at a flow rate of 1.0 mL/min using methanol/water (97:3 *v*/*v*) as mobile phase. Injection volume was 20 µL, and retention time was 9 min.

A calibration curve with acceptable linearity (R^2^ = 0.9998) was constructed by plotting the peak area versus dye concentration within 1–8 µg/mL concentration range. The relative standard deviation (RSD) of intra- and inter-day precision at three concentrations (1, 4, and 8 µg/mL) was less than 3%, and the accuracy ranged from 97.0% to 102.5% HPLC.

### 2.9. In Vitro Cell Studies

#### 2.9.1. Cell Culture

Human osteosarcoma U-2OS cells (ATCC, Manassas, VA, USA) were cultured in a humidified atmosphere at 37 °C, with 5% CO_2_, in DMEM medium (Invitrogen, Milan, Italy) supplemented with 1% *v/v* penicillin-streptomycin (Sigma-Merck, St. Louis, MO, USA) and 10% *v/v* fetal bovine serum (Sigma-Merck).

#### 2.9.2. Cytotoxicity

Cells were seeded in 96-well chemiluminescence plates and exposed for 72 h to the unloaded samples (NP_POS_2, NP_NEG_1, CaP-NP_POS_2B, CaP-NP_NEG_1B) at the same dilutions used for dye uptake experiments. Therefore, dilutions were expressed as theoretical Sudan Red III concentrations. The plates were treated with the ATPLite Luminescence Assay System kit (PerkinElmer, Waltham, MA, USA) and read via a chemiluminescence-based assay, using a Synergy HTX multi-plate reader (Bio-Tek Instruments, Winooski, VT, USA). The chemiluminescent units corresponding to the untreated cells were considered equal to 100% cell viability. The results were expressed as a percentage of chemiluminescence units (viability) of the treated cells relative to the chemiluminescence units (viability) of the untreated cells.

#### 2.9.3. Sudan Red III Cellular Uptake

Cells were seeded in 24-well plates and incubated 3 and 24 h with NPs loaded with 0.1–0.5–1.25–2.5–5-μg/μL of Sudan Red III. At the end of the incubation time, the culture medium was aspirated, the cells were washed twice with PBS and detached with 0.50 μL trypsin, and suspended in 250 μL of PBS. An aliquot of 50 μL was sonicated and used for the quantification of proteins using the BCA-1 kit (Sigma-Merck). The remaining cell suspension was transferred to a 96-well plate and used to read the intracellular fluorescence of Sudan Red III, as index of its uptake, with a Synergy HTX multi-plate reader. Excitation and emission wavelengths were 596 and 615 nm. The fluorescence units were converted into Sudan Red III nmoles, on the basis of a calibration curve carried out with free Sudan Red III solutions at the following concentrations: 1, 10, 100, 250, and 500 nmol/mL. The results were expressed as nmol/mg cellular proteins.

#### 2.9.4. Statistical Analysis

Results were analyzed via a one-way analysis of variance (ANOVA) and Tukey’s test, using GraphPad Prism software (v 6.01). *p* < 0.05 was considered significant. All data were expressed as means + SD.

## 3. Results and Discussion

The aim of this work was to develop CaP-coated NPs suitable to be tested for improving the treatment of different bone diseases. The strategy consists of using CaP as a coating for NPs to exploit its ability to bind to bone.

### 3.1. Preparation of NPs and Screening of Charging Agents

In the literature [12] CaP coated-1,2-dioleoyl-sn-glycero-3-phosphate (DOPA) liposomes were developed by adding in a single step calcium and phosphate ions to the liposome suspension; the formation of CaP coating was mainly due to the interaction of calcium ions with the negatively-charged phosphate groups of DOPA bilayers.

In the present work, a similar, properly adapted procedure was employed to coat NPs prepared with the “cold microemulsion dilution” technique. Since a net charge on the NP surface was essential to allow CaP coating, different charging agents were added to a reference µE, the composition of which has been extensively described in our previous work [20].

In such a way, both positively-charged (NP_POS_) and negatively-charged (NP_NEG_) uncoated NPs were prepared the composition of which is reported above (Table 1). Stearylamine was employed to obtain positively-charged NPs (NP_POS_1–2), while sodium TC or fatty acids such as myristic or stearic or behenic acid were used to confer a negative charge to the NPs, obtaining NP_NEG_2, NP_NEG_3, NP_NEG_4, and NP_NEG_5, respectively. Only in the case of NP_NEG_1 the negative charge of Epikuron^®^200 was exploited without any further addition of charging agent. For this purpose, the amount of Epikuron^®^200 was increased from 150 to 170 mg.

### 3.2. Characterization of NPs

#### 3.2.1. Particle Size and Zeta Potential Measurements

In Table 3 mean diameters and Zeta potentials of uncoated NPs are reported.

Considering the positively charged NPs (NP_POS_1 and NP_POS_2) it can be noted that mean size and Zeta potential depend on µE composition: indeed, the presence of TC in NP_POS_1 results in a lower value of Zeta potential (+5.01 mV) than NP_POS_2 (+34.03 mV), as the negatively-charged TC is supposed to partially neutralize the positive charge of stearylamine. Moreover, when benzyl alcohol was used as a cosurfactant, a significant reduction in mean size (205.6 vs. 370.9 nm) was observed. For this reason, NP_POS_2 have been selected for the following steps of the research.

Focusing on the negative NPs, probably some overlapping phenomena can occur determining the resulting NPs surface change as well as mean sizes. For instance, TC-Epikuron^®^200 interactions, which lead to the formation of mixed micelles, must not be underestimated: probably, TC locates at the interface and partially forms mixed micelles with Epikuron^®^200 removing it from the NP surface. This interaction might explain the less negative Zeta potential value noted for TC-containing NP_NEG_2 compared to NP_NEG_1, in which TC was not present and which had the highest amount of Epikuron^®^200. Therefore, NP_NEG_1 was the formulation with the most negative Zeta potential.

The introduction of fatty acids in µE composition determined a significant increase (up to 500 nm) in NPs mean size (NP_NEG_3–5) without any correlation with the fatty acid chain length. Moreover, less negative values of Zeta potential (about −12 mV) were observed.

On these bases, among all the developed systems, only NP_POS_2 and NP_NEG_1 were chosen to be coated with CaP, since they showed the smallest mean sizes and the most extreme surface charge.

#### 3.2.2. SEM Characterization

**Figure 1:**
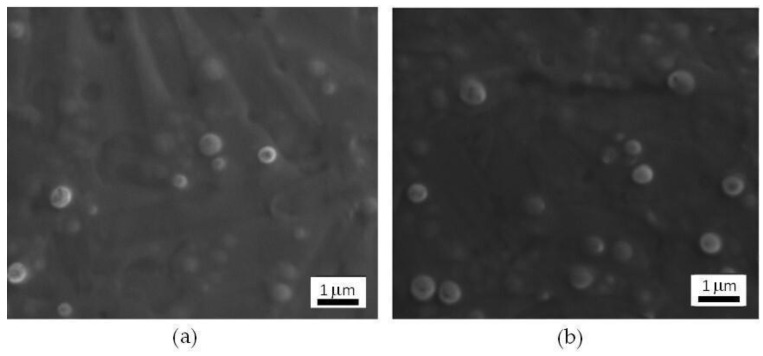
SEM micrographs of (**a**) NP_POS_2 and (**b**) NP_NEG_1.

The morphology of NP_POS_2 and NP_NEG_1 was studied by using SEM: both the positively-charged and the negatively-charged NPs were spherically shaped, appeared separated from each other and well-dispersed, confirming the lack of aggregation phenomena (Figure 1).

### 3.3. Coating Evaluation

A coating method to obtain CaP-coated NPs previously described in literature for liposomes [12] was properly adapted and tested (method A). This method was firstly applied to NP_POS_2: equal aliquots of 69 mM CaCl_2_ (calcium ion) and 41 mM H_3_PO_4_ (phosphate ion) aqueous solutions were simultaneously added to the unpurified NP_POS_2 suspension. The coating procedure was performed employing two different salts/NPs ratios, allowing NPs to react with the salt solutions for different times under magnetic stirring, as summarized in the above reported Table 2. In order to verify if CaP coating has occurred with method A, mean size and surface charge variations were monitored and the data are reported in Table 4.

As shown from DLS analysis, the size of all coated NP_POS_2A was more than twice than that of those uncoated, and the superficial charge was only slightly less positive than the initial one. The different NP/coating salts ratio neither affected the mean size nor the Zeta potential. The increase in mean size, more evident increasing the reaction time, shows that NPs have undergone some changes, but the obtained Zeta potential values by themselves did not demonstrate the formation of the coating onto NPs surface. For this reason, it was necessary to exploit different techniques to confirm the formation of NPs coating. Particularly, the following studies were focused on CaP-NP_POS_2A-3 as they showed the highest mean size and Zeta potential variations compared to the uncoated NP_POS_2.

**Figure 2:**
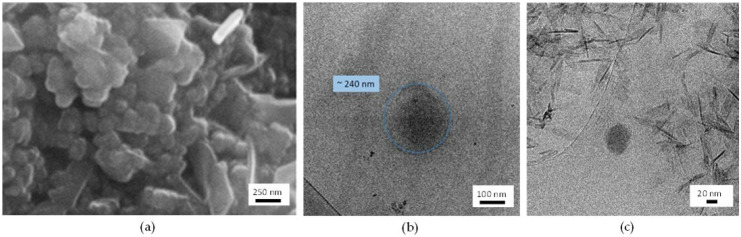
SEM micrographs of (**a**) CaP-NP_POS_2A-3 and TEM micrographs of (**b**) NP_POS_2 and (**c**) CaP-NP_POS_2A-3. SEM image collected at 20 kV with the standard SE detector. Instrumental magnification: (**a**) 15,000×, (**b**) 60,000×, and (**c**) 50,000×, respectively.

Hence, in order to detect the possible presence of the CaP coating, CaP-NP_POS_2A-3 were characterized by SEM and TEM and the results are summarized in Figure 2. Upon coating procedure (Figure 2a), evident aggregation of the globular NPs took place, probably due to inter-particle interactions between calcium and phosphate ions formed during the coating process. Moreover, the presence of crystals with elongated shape, which precipitated separately from the NPs, can be observed. Further TEM inspection put in evidence that the uncoated NP_POS_2 appears as a dark, poorly defined rounded spot, highlighted by the blue circle in Figure 2b, due to the low contrast with the grid on which the sample was placed. In addition, TEM image (Figure 2c) confirms that, for the CaP-NP_POS_2A-3 the coating was not effective. Indeed, NPs do not appear to be coated and the presence of elongated crystals, that are supposed to be calcium phosphate salts which precipitated separately, is clearly evidenced, in agreement with SEM analysis.

All of these results suggest that method A cannot be considered as an effective procedure of coating, since no formation of CaP-layer around NPs has been observed. Probably the method of liposome CaP-coating reported in the literature [12] is not suitable for coating the lipid systems under study. Our hypothesis is that the presence of surfactants/cosurfactants in NPs suspension, used in the preparation of the starting µE, can hinder and prevent the interaction between the calcium/phosphate salts and the lipid surface.

Therefore, a new coating method (method B) that includes a preliminary purification of NPs suspension, was developed, based on a previously reported layer-by-layer procedure [21]. Particularly, gel chromatography purification was employed to eliminate the excess surfactants/cosurfactants.

Successively, a series of dialysis steps was introduced to remove excess salts which resulted from the gel chromatography process. Moreover, CaCl_2_ and Na_2_HPO_4_ were added alternatively, in small amounts and in 1:1 molar ratio, in order to generate ion layers with opposite charge. After each addition, the mixture was magnetically stirred for 20 min to permit the interaction between the NPs surface and the salts. Na_2_HPO_4_ solution was lastly added to obtain negative-charged NPs able to interact with calcium ions, being calcium and phosphate the major minerals of the bone intercellular composite.

Different NPs/salts ratios were tested, but the best results in terms of mean size and Zeta potential, reported in Table 5, were obtained by adding serially different aliquots of salts to 3 mL of purified NPs.

**Figure 3:**
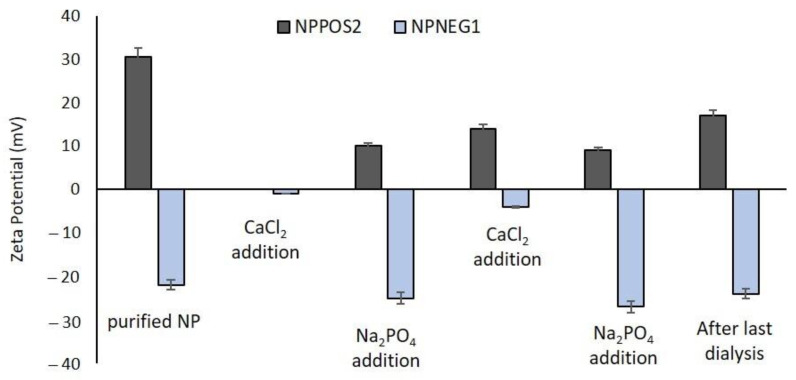
Variation of Zeta potential during coating process via method B.

DLS characterization of CaP-NP_POS_2B and CaP-NP_NEG_1B showed an increase of mean diameters for both types of coated NPs compared to the corresponding uncoated NPs, while Zeta potential values exhibit a different behavior. Indeed, while CaP-NP_NEG_1B showed a negative surface charge, probably due to the phosphate groups which remain exposed to the outer NPs surface, the Zeta potential of CaP-NP_POS_2B was always positive, although the value is decreased compared to uncoated NP_POS_2. In Figure 3 the variation of Zeta potentials during the coating processes is reported.

As shown, an evident change in Zeta potential values occurred upon each salt addition. Indeed, after the addition of Na_2_HPO_4_ solution, the Zeta potential of NP_POS_2 decreased from about +30 mV to +10 mV and then increased to +15 mV after CaCl_2_ solution introduction and finally decreased again up to +8 mV after the last addition of Na_2_HPO_4_ solution. The final value of Zeta potential after removal of excess salts by dialysis process was +17 mV.

The alternation of Zeta potential values upon calcium and phosphate salts addition is more evident for NP_NEG_1. It increased to near zero values after CaCl_2_ addition and returned to about −25 mV after Na_2_HPO_4_ addition. This trend could indicate the progressive deposition of the added ions around the NPs.

**Figure 4:**
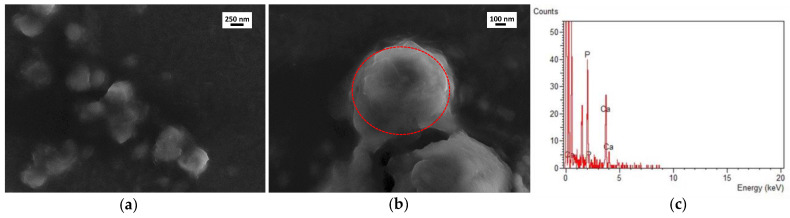
FESEM micrographs of CaP-NP_NEG_1B at different magnifications (**a**) 5000×, (**b**) 10,000×. Panel (**c**) shows the EDS spectrum of the region highlighted in (**b**) by the red dashed circle. FESEM images collected at 25 kV with the standard SE detector.

**Figure 5:**
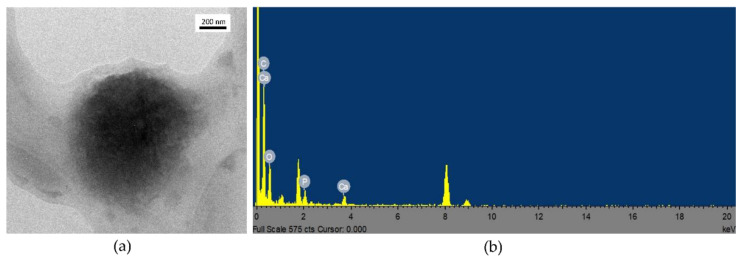
TEM image of (**a**) CaP-NP_NEG_1B and (**b**) EDS spectrum of the region displayed in (**a**). The peaks around 8 KeV are due to the presence of Cu in the grid. Instrumental magnification: 15,000×.

However, these results alone do not demonstrate the presence of CaP layers onto NPs surface. To further verify such hypotheses, SEM and TEM measurements were performed on the CaP-NP_NEG_ series. As an example, Figure 4 and Figure 5 summarize the results obtained for the CaP-NP_NEG_1B sample.

NPs of the CaP-NP_NEG_1B sample expose irregular and rough surfaces, and this is particularly evident in Figure 4b. This feature can be an indication of the presence of a coating layer. The same remark is valid for CaP-NP_POS_2B in which the surface of the NPs looks the same (images not reported). EDS analyses performed on both positive, reported in Figure S1 of the Supplementary Materials, and negative CaP-NPs, shown in Figure 4c, put in evidence the presence of calcium in both cases, which suggests that the coating observed via FESEM is possibly calcium phosphate-based. However, the intensity of Ca-peaks in Figure 4 is greater than that of Ca-peaks in Figure S1 indicating a low CaP-coating formation on NP_POS_2 surface.

Similarly, TEM characterization confirmed that the NPs of the CaP-NP_NEG_1B sample appear darker in contrast than the uncoated ones, see for example Figure 2b, and embedded within smaller particles (Figure 5). Again, the EDS analysis detected the presence of calcium, instead absent in NP_NEG_1 (Figure S2).

**Figure 6:**
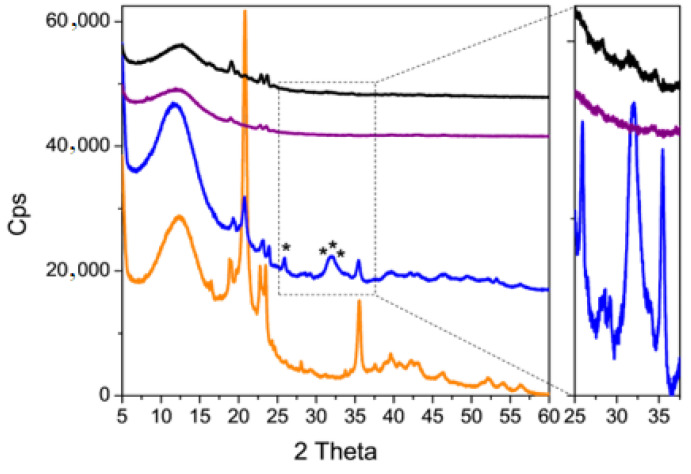
XRD patterns of NP_NEG_1 (orange line), CaP-NP_NEG_1B (blue line), NP_POS_2 (purple line) and CaP-NP_POS_2B (black line). Zoom: comparison of the patterns in the selected region. The asterisks * highlight the position of the peaks assigned to hexagonal hydroxyapatite.

XRD measurements were carried out to further investigate the nature of the CaP coating and the changes of the microstructure upon coating, the results are shown in Figure 6. Upon coating procedure, an overall reduction in intensity of the peaks related to crystalline structure of the lipid NPs is observed (blue line vs. orange line). It is well-known that the crystalline structure related to the chemical nature of lipid NPs strongly affects the drug delivery behavior of the carrier system in determining whether the drug would be delivered or tightly incorporated [22]. Indeed, lipid NPs with a less ordered arrangement increase the drug loading capacity [23]. Moreover, a narrow peak at 2θ equal to 25.9° and a broad peak, around 2θ equal to 32°, which result from the overlapping of the XRD patterns, are observed in CaP-NP_NEG_1B sample (marked with asterisks in Figure 6, blue line) after coating. These peaks can be assigned to the 002, 211, 112, and 300 planes of hexagonal hydroxyapatite (JCPDS file number 00-009-0432). Interestingly, the main broad peak of hydroxyapatite is also detected in the pattern collected on the CaP-NP_POS_2B sample (black line) and it is not observed on the uncoated NP_POS_2 (purple line). Nevertheless, the intensity of this peak resulted much lower in the black pattern than that in the blue one, further supporting our hypothesis that NP_NEG_1 are more CaP-coated than NP_POS_2.

**Figure 7:**
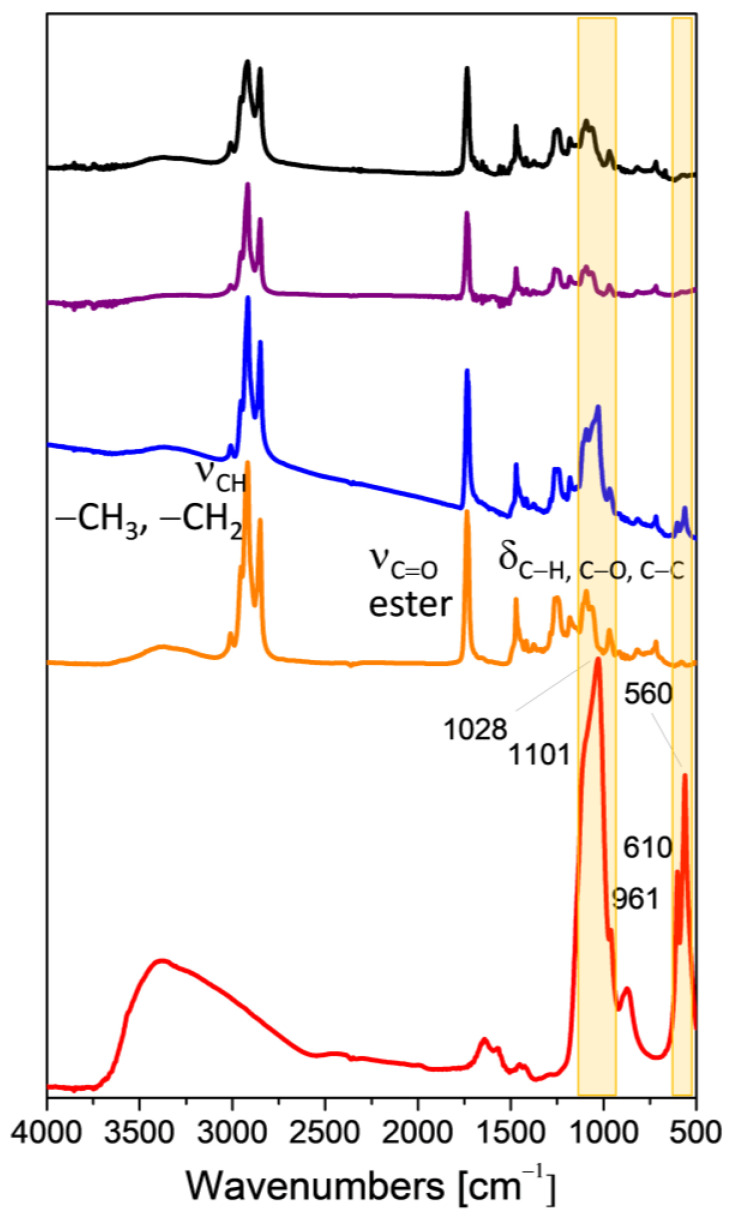
FTIR absorbance spectra of Ca_3_PO_4_ (red line), NP_NEG_1 (orange line), CaP-NP_NEG_1B (blue line), NP_POS_2 (purple line), and CaP-NP_POS_2B (black line).

In order to follow the formation of the CaP coating on the NPs, CaP-NP_NEG_1B, and CaP-NP_POS_2B, as well as the respective uncoated samples were characterized by FTIR spectroscopy. Indeed, some absorption bands may disappear and some other bands may appear during the chemical reaction among the compounds present in the formulation. The comparison between the FTIR spectra of CaP-NP_NEG_1B (blue line) and CaP-NP_POS_2B (black line) is reported in Figure 7. The spectra of Ca_3_PO_4_ (red line), of NP_NEG_1 (orange line), and of NP_POS_2 (purple line) are also reported as reference samples. On one hand, the spectrum of Ca_3_PO_4_ (red line) is characterized by strong bands at 1101 and 1028 cm^−1^, that are due to the components of the triply degenerate v_3_ antisymmetric P-O stretching mode. The band at 961 cm^−1^ can be assigned to the non-degenerate P-O symmetric stretching v_1_ mode. Moreover, the bands at 610 and 570 cm^−1^ are assigned to the components of the triply degenerate v_4_ O-P-O bending mode [24]. On the other hand, the spectra of NPs displays intense and narrow peaks in the 3000–2750 cm^−1^ range bands due to aliphatic stretching mode of C–H bond, strong band at 1732 cm^−1^ assigned to the stretching vibration of the C=O ester, at 1466 cm^−1^ due to CH_2_ scissoring [25], at 1260 and 956 cm^−1^ related to the OH bending vibration, at 1089 cm^−1^ ascribed to skeletal vibration, and at 720 cm^−1^ due to the rocking and bending vibration modes typical of aliphatic chains.

Upon CaP coating, a new intense component at 1028 cm^−1^ along with bands at 961, 610 and 560 cm^−1^ are observed in the spectrum of the CaP-NP_NEG_1B sample (blue line, highlighted by the yellow bars as eye guides). These absorptions are present, even if quite lower in intensity, also in the case of the CaP-NP_POS_2B sample (black line) and are not observed in the spectrum of the uncoated NPs-NP_NEG_1 (orange line) and NP_POS_2 (purple line). These spectroscopic features further confirm the presence of CaP coating on the NPs.

### 3.4. Stability Studies

**Figure 8:**
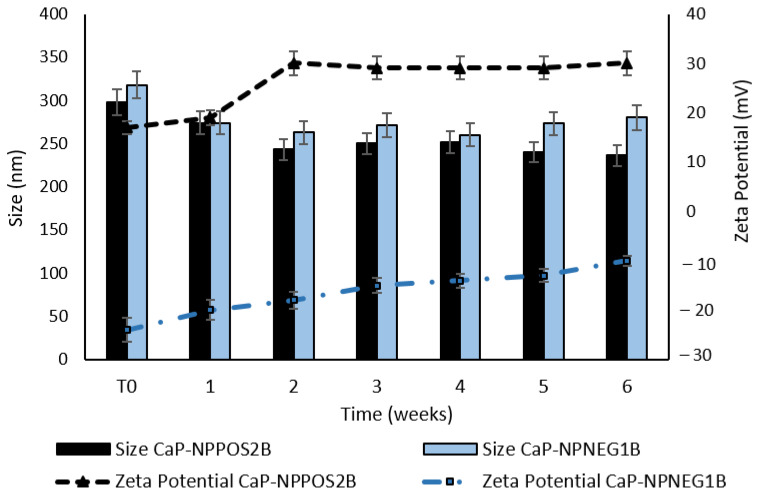
Overtime evolution of mean diameters and Zeta potential values of CaP-NP_POS_2B and CaP-NP_NEG_1B.

In order to study the overtime stability of CaP-NP_POS_2B and CaP-NP_NEG_1B, mean diameters (DLS) and Zeta potential measurements were performed for six weeks on samples stored at 4 °C. In Figure 8 the overtime evolution of both parameters is reported.

As shown in Figure 8, a slight decrease in mean sizes and also a certain change in Zeta potential values were noted in both the NP samples. Namely, compared to the fresh samples, CaP-NP_POS_2B showed a higher Zeta potential (+19 mV) after one week and became much more positive (+30 mV) after two weeks. Similarly, Zeta potential values of CaP-NP_NEG_1B gradually increased from −24 mV to −10 mV in six weeks.

It is probable that the interactions between the NP surface charge and the coating ions decreased, determining a loss of the CaP coating overtime and resulting in a mean size reduction and in a gradual rise of the Zeta potentials. Accordingly, to improve CaP-NPs stability, limiting CaP loss from NPs surface, they were freeze-dried in the absence or in the presence of different cryoprotectants. In Table 6, mean diameters and Zeta potentials of freshly prepared and re-suspended freeze-dried samples are reported.

As evident, the presence of cryoprotectants is necessary to obtain samples with characteristics similar to those of the freshly prepared NPs. Indeed, after resuspension both the coated NP types (CaP-NP_POS_2B and CaP-NP_NEG_1B) showed higher mean sizes (more than 1 µm) compared to the fresh samples, while, when cryoprotectants were added, NP mean diameters were comparable to the initial ones. Also, Zeta potential values were maintained after freeze-drying. No evident differences were noted between the two cryoprotectants tested. These results indicated the maintenance of CaP coating around the NP and the suitability of the freeze-drying process to increase the CaP-NPs stability overtime.

### 3.5. Incorporation of Sudan Red III and Determination of Entrapment Efficiency

As previously mentioned, Sudan Red III was used as a model lipid molecule to study the CaP-NP entrapment capability and to perform in vitro cellular uptake tests. In Table 7 the values of %EE of Sudan Red III in either uncoated or coated NPs are reported.

As shown, %EE of Sudan Red III in uncoated NPs was very high (91.2% for NP_POS_2 and 97.4% for NP_NEG_1) but it decreased by about 20% after CaP coating, probably due to the numerous agitation and dialysis processes to which the NPs are subjected.

The release study further confirmed the entrapment of Sudan Red III (see Supplementary Materials-Figure S3).

### 3.6. In Vitro Cell Studies

#### 3.6.1. Cytotoxicity

**Figure 9:**
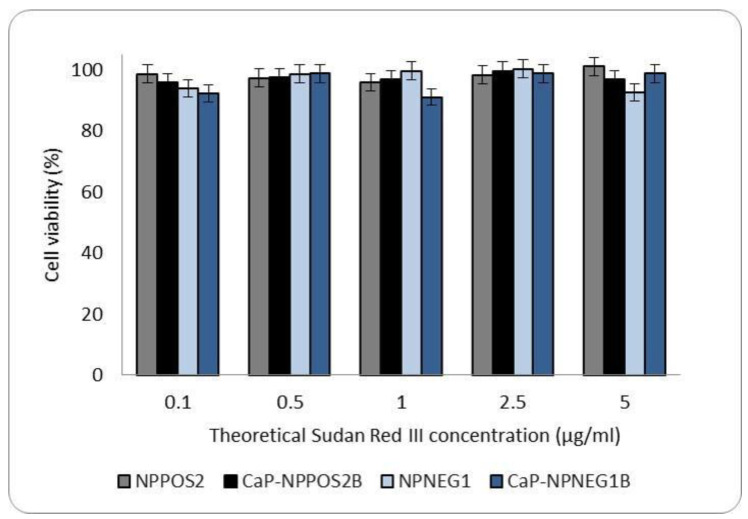
Osteosarcoma cell viability after incubation with NPs. Data are expressed as mean + SD (*n* = 3).

U-2OS osteosarcoma cells were exposed dose-dependently to NP_POS_2, CaP-NP_POS_2B, NP_NEG_1 or CaP-NP_NEG_1B and the viability was measured after 72 h. As shown in Figure 9, none of the formulations significantly decreased cell viability at any concentration tested, indicating a good biocompatibility.

#### 3.6.2. Sudan Red III Cellular Uptake

**Figure 10:**
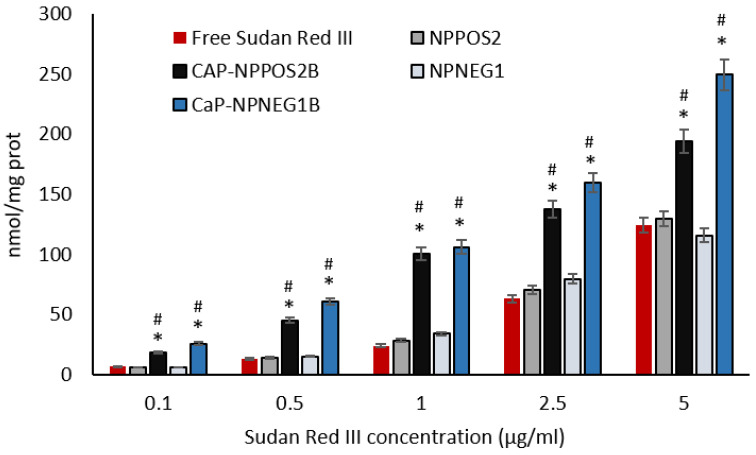
Uptake of Sudan Red III in human osteosarcoma cells after 24 h. Data are expressed as mean + SD (*n* = 3), * *p* < 0.05 vs. free Sudan Red III; ^#^ *p* < 0.05 vs. NP_POS_2/NP_NEG_1.

To evaluate the potential delivery of a lipid entrapped substance into osteosarcoma cells, we assessed the intracellular content of Sudan Red III loaded into the different NPs, as an index of intracellular uptake, in cells incubated for 24 h (Figure 10). The intracellular delivery of the dye was concentration-dependent, for both free Sudan Red III and Sudan Red III loaded in NPs. At all the tested concentrations the delivery of the dye by the uncoated NPs was similar to that of the free dye, suggesting that uncoated NPs did not provide an advantage. No appreciable differences were noticed between negatively-charged and positively-charged NPs. On the contrary, both negatively-charged and positively-charged NPs CaP-coated NPs delivered significantly more Sudan Red III than uncoated NPs or free dye. This result indicates that CaP coating confers a strong advantage in delivery entrapped substances within osteosarcoma cells.

Such CaP-coated NPs will be object of a soon forthcoming future investigation regarding drug entrapment, in vitro release, and osteosarcoma cell uptake.

## 4. Conclusions

The main purpose of this work was to develop CaP-coated lipid NPs suitable to improve the treatment of different bone diseases. In particular, starting from a liposome coating method reported in the literature, an innovative CaP coating method was successfully developed to obtain novel CaP-coated lipid NPs. More specifically, NPs were prepared by the technology called “cold dilution of microemulsions”. Modulating the composition of the starting µE, it was possible to obtain NPs with differently charged surfaces, serving as attaching points for the coating salts that precipitated layer-by-layer.

The main potential advantage of the novel developed lipid CaP-coated NPs is their higher uptake by osteosarcoma cells than uncoated ones, suggesting their selectivity to bone tissue as a promising prerequisite for bone-targeted drug delivery.

Several physico-chemical techniques were then employed to characterize the obtained CaP coating. Additionally, the freeze-drying process was introduced to increase the overtime stability of the CaP-NPs, preventing the loss of the coating from the NP surface.

In vitro cell viability studies performed on the developed systems evidenced no cytotoxicity on osteosarcoma cells for all the tested samples. Furthermore, the loading of Sudan Red III into CaP-NPs, used as a model, mimicking an entrapped drug, allowed to evaluate the dye uptake in human osteosarcoma cells. The results highlighted that CaP-NPs delivered more dye to the cells within 24 h than the uncoated NPs and showed that the CaP-coating plays a key role in enhancing the delivery of an entrapped molecule to bone cells.

However, further studies are needed to assess the possible employment of the here proposed CaP-NPs as a selective drug delivery system for bone therapies. In the near future, antitumor drugs will be loaded into CaP-NPs to study their potential application in human and canine osteosarcoma treatment.

## Figures and Tables

**Table 1:** Composition of µEs and corresponding NPs prepared with different charging agents.

		Ingredients (mg)	µE_POS_1/ NP_POS_1	µE_POS_2/ NP_POS_2	µE_NEG_1/ NP_NEG_1	µE_NEG_2/ NP_NEG_2	µE_NEG_3/ NP_NEG_3	µE_NEG_4/ NP_NEG_4	µE_NEG_5/ NP_NEG_5
**NPs**	µEs	Trilaurin	60	60	60	60	60	60	60
		s-EA	200	200	200	200	200	200	200
		Epikuron^®^200	150	150	170	150	150	150	150
		Cremophor^®^RH60	50	50	50	50	50	50	50
		Propylene glycol	200	200	100	-	200	200	200
		Benzyl alcohol	-	40	-	-	20	20	20
		TC	30	-	-	10	-	10	20
		Myristic acid	-	-	-	-	10	-	-
		Stearic acid	-	-	-	-	-	10	-
		Behenic acid	-	-	-	-	-	-	10
		Stearylamine	10	10	-	-	-	-	-
		s-Water	700	700	700	700	700	700	700
		Dilution water (mL)	5	5	5	5	5	5	5

**Table 2:** Operating conditions employed in the method A of CaP coating.

Conditions	NP_POS_2A-1	NP_POS_2A-2	NP_POS_2A-3	NP_POS_2A-4	NP_POS_2A-5
69 mM CaCl_2_ (mL)	1	1	1	1	1
41 mM H_3_PO_4_ (mL)	1	1	1	1	1
NP_POS_2 (mL)	1	1	1	0.5	0.5
Reaction time (min)	120	180	240	180	240

**Table 3:** Mean diameters and Zeta potential values of uncoated NPs.

Samples	Mean Diameter (nm) ± S.E. (P.I)	Zeta Potential (mV) ± S.E.
NP_POS_1	370.9 ± 5.3 (0.271)	+5.01 ± 1.91
NP_POS_2	205.6 ± 3.9 (0.199)	+34.03 ± 3.90
NP_NEG_1	250.5 ± 0.5 (0.235)	−23.54 ± 1.79
NP_NEG_2	252.1 ± 4.5 (0.182)	−15.63 ± 2.42
NP_NEG_3	538.4 ± 33.0 (0.259)	−11.94 ± 2.47
NP_NEG_4	511.0 ± 12.3 (0.190)	−12.07 ± 2.00
NP_NEG_5	524.7 ± 4.2 (0.258)	−12.98 ± 0.95

**Table 4:** Mean diameters and Zeta potential values of NP_POS_2, uncoated and coated via method A.

Samples	Mean Diameter (nm) ± S.E. (P.I)	Zeta Potential (mV) ± S.E.
NP_POS_2	205.6 ± 3.9 (0.199)	+34.03 ± 3.90
CaP-NP_POS_2A-1	481.7 ± 9.8 (0.308)	+25.90 ± 1.24
CaP-NP_POS_2A-2	488.5 ± 15.2 (0.338)	+26.44 ± 1.35
CaP-NP_POS_2A-3	502.8 ± 14.2 (0.299)	+21.28 ± 3.55
CaP-NP_POS_2A-4	445.4 ± 25.0 (0.387)	+25.30 ± 2.02
CaP-NP_POS_2A-5	455.9 ± 7.5 (0.372)	+24.03 ± 2.88

**Table 5:** Mean diameters and Zeta potential values of NP_POS_2 and of NP_NEG_1, uncoated and coated via method B.

Samples	Mean Diameter (nm) ± S.E. (P.I)	Zeta Potential (mV) ± S.E.
NP_POS_2	205.6 ± 3.9 (0.199)	+34.03 ± 3.90
CaP-NP_POS_2B	298.7 ± 10.3 (0.284)	+17.61 ± 1.72
NP_NEG_1	250.5 ± 0.5 (0.235)	−23.54 ± 1.79
CaP-NP_NEG_1B	318.2 ± 11.6 (0.278)	−23.43 ± 1.82

**Table 6:** Mean diameters and Zeta potential values of fresh and freeze-dried samples.

Samples		Mean Diameter (nm) ± S.E.	Zeta Potential (mV) ± S.E.
CaP-NP_POS_2B	fresh	298.7 ± 10.3	+17.61 ± 1.72
CaP-NP_POS_2B	freeze-dried	1040 ± 20.3	+20.15 ± 2.7
CaP-NP_POS_2B + trehalose	freeze-dried	320.3 ± 9.5	+21.37 ± 2.5
CaP-NP_POS_2B + glucose	freeze-dried	305.5 ± 8.9	+19.56 ± 3.19
CaP-NP_NEG_1B	fresh	318.2 ± 11.6	−23.43 ± 1.82
CaP-NP_NEG_1B	freeze-dried	1011.8 ± 97.9	−19.47 ± 2.38
CaP-NP_NEG_1B + trehalose	freeze-dried	343.5 ± 10.0	−20.42 ± 2.16
CaP-NP_NEG_1B + glucose	freeze-dried	322.0 ± 16.8	−24.19 ± 1.77

**Table 7:** Sudan Red III entrapment efficiency (%EE) in NPs.

Samples	%EE
NP_POS_2	91.2 ± 3.5
CaP-NP_POS_2B	75.6 ± 2.7
NP_NEG_1	97.4 ± 3.3
CaP-NP_NEG_1B	77.5 ± 2.4

## Data Availability

The data presented in this study are available on request from the corresponding author.

## Supplementary Materials

The following are available online at https://www.mdpi.com/article/10.3390/nano11112983/s1. Figure S1: EDS spectrum collected on the CaP-NPPOS2B sample; Figure S2: EDS spectrum collected on NPNEG1; Figure S3: Sudan Red III release profiles.
