# Supplementary material for: Calcium Phosphate-Coated Lipid Nanoparticles as a Potential Tool in Bone Diseases Therapy
Source: Nanomaterials (Basel). 2021 Nov 6;11(11):2983. doi: 10.3390/nano11112983 (PMC8625061; doi:10.3390/nano11112983)
Supplement: Supplementary file 1 [file nanomaterials-11-02983-s001.zip › nanomaterials-1406603-supplementary.pdf]

Supplementary materials

Calcium Phosphate-Coated Lipid Nanoparticles as a Potential Tool in Bone Diseases Therapy

Simona Sapino [1], Giulia Chindamo [1], Daniela Chirio [1,*], Maela Manzoli [1], Elena Peira [1], Chiara Riganti, [2,*] and Marina Gallarate [1]

[1] Department of Drug Science and Technology, University of Torino, via P. Giuria 9, 10125 Torino, Italy; simona.sapino@unito.it (S.S.); giulia.chindamo@unito.it (G.C.); maela.manzoli@unito.it (M.M.); elena.peira@unito.it (E.P.); marina.gallarate@unito.it (M.G.)

[2] Department of Oncology, University of Torino, via Santena 5/bis, 10126 Torino, Italy

* Correspondence: daniela.chirio@unito.it (D.C.); chiara.riganti@unito.it (C.R.); Tel.: +39-011-6707167 (D.C.); +39-011-6705857 (C.R.)

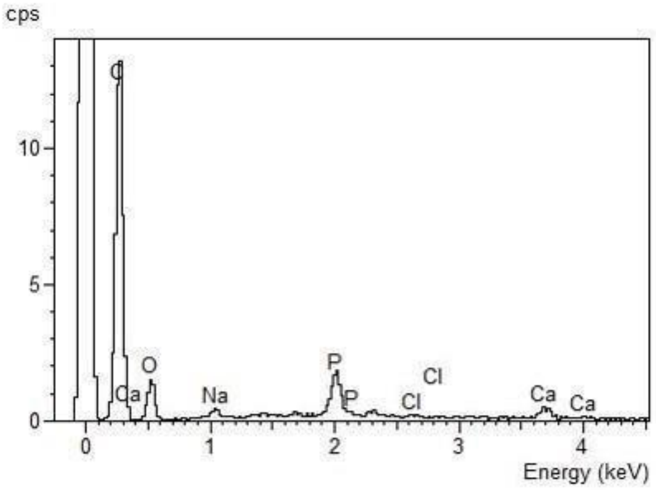

Figure S1. EDS spectrum collected on the CaP-NP_{POS}2B sample.

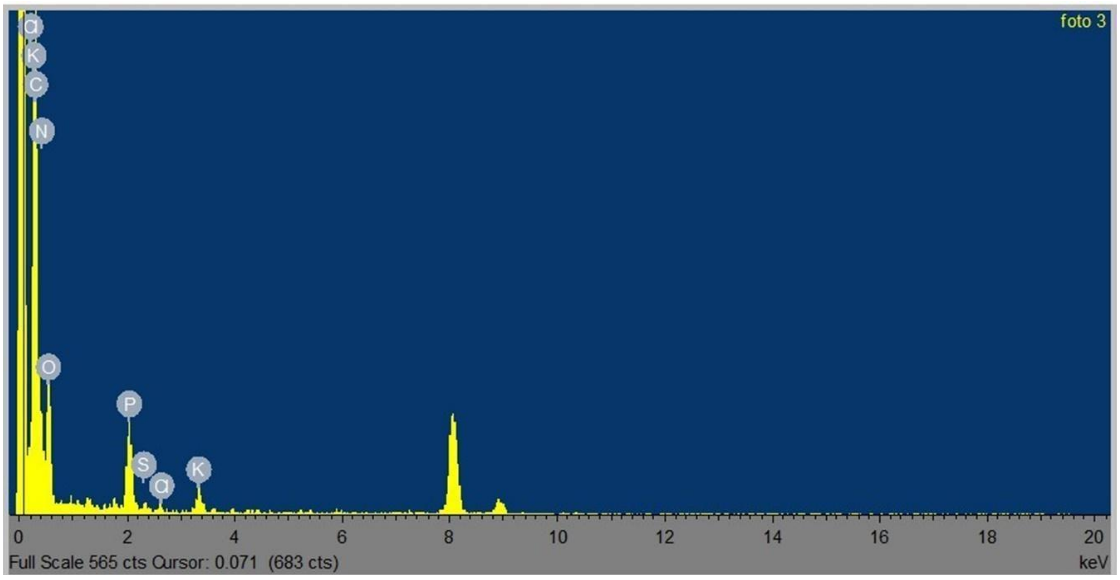

Figure S2. EDS spectrum collected on NP_{NEG}1. The peaks around 8 KeV are due to the presence of Cu in the grid.

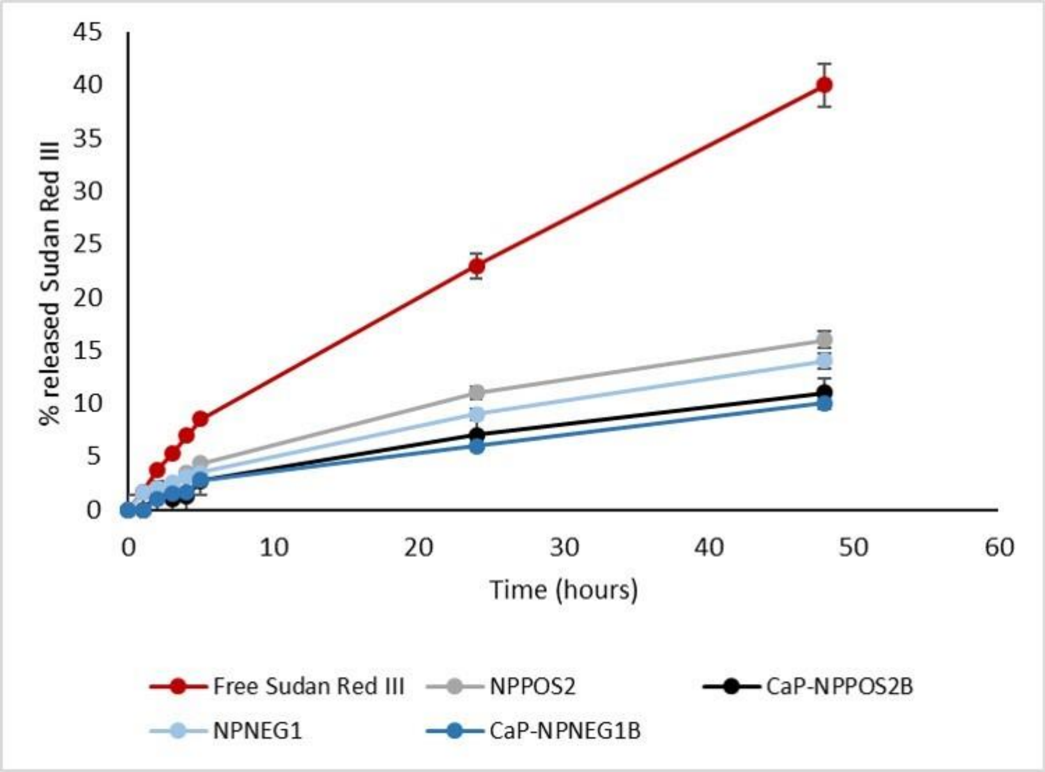

Figure S3. Sudan Red III release profiles.

Sudan Red III release study

Method: The in vitro release of Sudan Red III from NPs was determined by the nonequilibrium dialysis method using a multicompartmental rotating cell system consisting of donor and receptor compartments of equal volume (1.5 mL) separated by a dialysis membrane (cut off 14000 Da).

The receiving medium was 20% w/w ethanol in water. An aqueous suspension of Free Sudan Red III and dye loaded NPs ($NP_{POS}2$, CaP-$NP_{POS}2B$, $NP_{NEG}1$ and CaP-$NP_{NEG}1B$) were used as donor formulations in separate experiments. At fixed times, the receptor solution was withdrawn, and the compartment was refilled with fresh receiving medium, obtaining sink conditions. The Sudan Red III concentration in the receiving medium was determined by HPLC.

Result: No burst effect was observed from neither coated nor from uncoated NPs, suggesting an effective incorporation of the dye in the lipid matrix. The release profiles of Sudan Red III suspension and of dye-loaded NPs were quite different: Sudan Red III diffused faster from the dye suspension then from NPs formulations, without any evident differences between coated and uncoated NPs .

Therefore, the in vitro release results confirmed the dye entrapment in NPs and proved the suitability of these NPs as potential drug delivery system.
